# ﻿*Festucasilana* (Poaceae), a new species from the Sila plateau in Italy

**DOI:** 10.3897/phytokeys.255.146942

**Published:** 2025-04-10

**Authors:** Mattia Pallanza, Simone Orsenigo, Nicola Maria Giuseppe Ardenghi, Liliana Bernardo, Petr Šmarda, Petra Šarhanová, Graziano Rossi, Bruno Foggi

**Affiliations:** 1 Department of Earth and Environmental Sciences, University of Pavia, Via Sant'Epifanio 14, 27100 Pavia, Italy; 2 National Biodiversity Future Centre, 90133 Palermo, Italy; 3 Botanic Garden, University Museum System, University of Pavia, Via Sant'Epifanio 14, 27100 Pavia, Italy; 4 Department of Biology, Ecology and Earth Sciences, University of Calabria, Via Pietro Bucci, 87030 Arcavacata di Rende (Cosenza), Italy; 5 Department of Botany and Zoology, Faculty of Science, Masaryk University, Kotlářská 2, 61137 Brno, Czech Republic; 6 Department of Biology, University of Florence, Via G. La Pira 4, 50121 Firenze, Italy

**Keywords:** Calabria, endemic, fescue, flow cytometry, Italy, morphometry, new species, ploidy, RAD sequencing

## Abstract

A new hexaploid species of fine-leaved fescue from Festucasect.Festuca (*Festucasilana* Ardenghi, Pallanza & Foggi, **sp. nov.**) endemic to the Sila plateau is described. The new taxon shows morphological affinities with species of the *F.marginata* group from which it can be distinguished by higher ploidy, ecology, and leaf cross section anatomy. ddRADseq data suggests *F.silana* could be a local hexaploid descendant/derivate of the widespread diploid *F.marginata*. Its distribution is restricted to several localities in the Sila highlands of the Southern Apennines, Italy.

## ﻿Introduction

*Festuca* L. (Poaceae, Loliinae) is one of the most species-rich genera of grasses (Poaceae), containing worldwide around 680 accepted species (Govaerts 2024). Within the *Festuca* genus, “fine-leaved fescues” represent the most important group, with approximately 450 species (Foggi and Tison 2014; Kellogg 2015). The taxonomy and systematics of *Festuca* has historically been regarded by botanists as difficult, due to the morphological convergence of unrelated taxa, phenotypic plasticity, complex nomenclature and a lack of clear universally accepted diagnostic features between taxa. The first solid foundation to modern taxonomic *Festuca* studies was posed by Eduard Hackel in his “*Monographia Festucarum Europaearum*” (Hackel 1882). Hackel introduced novelties in the approach to identification and classification of fescues, including the study of the leaf cross-section, one of the main investigation tools still in use in modern festucology. Since then, many scholars have dedicated their efforts toward the taxonomy and systematics of *Festuca*, integrating the initial morphometrical approach with more modern techniques stemming from technologicals and scientific advances. These include analysis of the relative genome size (Šmarda et al. 2008; Martínez-Sagarra et al. 2021), chromosome number (Šmarda and Kočí 2003; Ardenghi et al. 2016), DNA sequencing (Boeuf et al. 2022, Kriuchkova et al. 2023, Mucko et al. 2024) and electron microscopy (Ortúñez and Cano-Ruiz 2013). Although the implementations of these new methods emanate from improved floristic records and checklist (Roleček et al. 2019; Gudkova et al. 2023; Bednarska et al. 2024), to the conservation and applications of the taxa studied (Ardenghi et al. 2017), most recent works have focused on groups of species, using an integrated approach combining multivariate morphometry, ploidy level and genetics (Šmarda et al. 2007; Foggi et al. 2012; Ardenghi et al. 2024; Bednarska et al. 2024).

During an extensive sampling conducted across the European Mediterranean basin in 2014 by N.M.G. Ardenghi, an unusual taxon belonging to the fine-leaved fescues was collected in different localities of the Sila plateau (Italy). The entity was already known and reported by Sarfatti in the “Prodoromo della Flora della Sila” under FestucaovinaL.subsp.laevisHack.var.gallica(Hack.) St.-Yvessubvar.costei St.-Yves (≡ *Festucacostei* (St.-Yves) Markgr.-Dann.) based on the original identification by Markgraf-Dannenberg (Sarfatti 1959). However, this taxon has been overlooked until now. A preliminary study of morphology and leaf cross section anatomy of both fresh samples and herbaria specimens including the holotype for *F.costei*, led us to question the identification proposed by Markgraf-Dannenberg. In fact, these fescues differ from *F.costei* in a series of readily identifiable features, especially in the leaf cross-section (e.g. number of vascular bundles, development and organization of the sclerenchyma, etc.) and rather appear to be morphologically in between the *F.stricta* and the *F.marginata* groups. In particular, the Sila entity appears to have the typical cross sections of the former and the overall morphology of the latter.

Due to the unusual combination of leaf cross-section anatomy and general morphology, we initially thought it could be a taxon of the *F.stricta* group (i.e.: *F.stricta* Host, *F.rupicola* Heuff., *F.trachyphylla* (Hack.) R.P.Murray) which present similar patterns in the organization of the tiller leaves sclerenchyma. This is especially true for *F.trachyphylla*, which is also the only species from the stricta group to normally present 7 vascular bundles in the cross section, a shared characteristic with the Sila specimens. Nevertheless, the samples still share numerous morphological similarities (e.g. smooth leaf blades, glabrous spikelets, etc.) with *Festucamarginata* (Hack.) K. Richt., which occurs in similar types of habitats across Italy.

To better understand the relationships among these different taxa, we chose to apply an integrated approach combining classical morphometry, ploidy level analysis using flow cytometry and ddRADseq sequencing. The results from these combined analyses support that the taxon from the Sila plateau is a new endemic species.

## ﻿Materials and methods

### ﻿Materials

Overall, 177 individuals representing 25 populations of five different taxa (*F.marginata*, *F.rupicola*, *F.stricta*, *F.trachyphylla* and the proposed new species) ranging from the Alps to the Apennines and from fresh and herbarium material (BRNU, FI, G, MSNM, PAV; Herbaria codes follow Thiers 2024, updated continuously) were morphologically studied (Suppl. material 1: tables S4, S5). Specimens were carefully selected to ensure a balanced representation of the different taxa. Fifty of the freshly collected individuals from the same populations were also analyzed using flow cytometry.

### ﻿Morphometric analysis

We selected 35 morphological characters for the analysis (Table 1), choosing those considered as diagnostic for fine-leaved fescues.

**Table 1.** Morphological characters used in multivariatue analyses and their coding. QD = quantitative discrete, QC = quantitative continuous, BI = binary, CO = ordinal.

Code	Character	Type
Clm_L	Culm length	QC
LfB_L	Tiller leaf blade length	QC
Pan_L	Panicle length	QC
Pan_Sk	Panicle scabridity	CO
Pan_Pb	Pubescence at the base of the panicle	CO
Clf_L	Culm leaf blade length	QC
Sh_Nd	Sheath to node distance	QC
InfBr_L	Inferior branch of the panicle length	QC
TilSh_Pb	Tiller sheaths pubescence	CO
Nd_Pr	Node pruinosity	BI
Sh_Pr	Tiller sheaths pruinosity	BI
Sh_Col	Tiller sheaths color	CO
Sk_Deg	Tiller leaves scabridity degree	CO
Sk_Ext	Percentage of tiller leaves scabrid surface	QC
Sc_Org	Sclerenchyma organization	CO
Sc_CT	Central sclerenchyma strand thickness	CO
Sc_MT	Marginal sclerenchyma strands thickness	CO
KlMR	Keel to middle rib distance	QC
Klmg	Keel to margins distance	QC
VB_N	Number of vascular bundles	QD
R_N	Number of accessory ribs	QD
Shp	Leaf cross-section outline shape	CO
Ep_Und	Presence of epidermal undulations	BI
Ep_Ind	Richness of epidermal indumentum	CO
LH	Longest hair in the abaxial surface of the cross-section	QC
Sp_L	Spikelet length	QC
LGl_L	Lower glume length	QC
UGl_L	Upper glume length	QC
UGl_hW	Upper glume half-width	QC
Lm_L	Lemma length	QC
Lm_hW	Lemma half width	QC
A_L	Awn length	QC
Gl_Pb	Glumes pubescence	CO
Lm_Pb	Lemma pubescence	CO
Sp_Sh	Spikelet shininess	CO

Measurements were performed according to the standards described in Foggi 1999 (which complies with Hackel 1882; Saint-Yves 1913; Ellis 1976; Wilkinson and Stace 1991) with minor modifications. To account for intraindividual variation, we chose to either keep the highest recorded value for the sample or to use the mean value calculated from three different measurements depending on the morphological character. Characters concerning leaf cross sections follow a design similar to Bednarska et al. (2024). Spikelet, floret and other microscopic characters were observed under a Eurotek NB-50T stereomicroscope at magnifications of 8x–10x. Leaf cross sections were studied with a Carl Zeiss Axiostar Plus microscope at magnifications of 40x–100x. Both microscopes were coupled with a camera (ToupTek USB3.0 Eyepiece Camera S3CMOS05000KPA) and all observed characters were measured using Toupview ver. 4.11.19728.20211022 software.

Each quantitative character was tested for normality of distribution within taxa using the Shapiro-Wilk test. Some characters had non-normal distribution for some taxa, however, since the chosen analyses have been shown to be robust to violation of the normality of distribution assumption (Klecka 1980), we decided to continue without transforming the data. To further support this decision, it has to be clear that non-fitted variables would have to be transformed as a whole, independently from the taxon, leading to a weakened perception of the actual morphological variability among taxa. Characters were also tested for significant correlations (>0.95) via Spearman’s non-parametric coefficient. High correlation was found only between characters related to the tiller leaves’ scabridity (Sk_Deg, Sk_Ext, Ep_Ind). Among them, we decided to keep only the density of the abaxial epidermis indumentum as observed in the cross section (Ep_Ind) in the analyses as it was the least subjective to measure. We also decided not to include the culm length and tiller leaf blade length (Clm_L and LfB_L respectively) due to their high dependency on environmental factors such as grazing, trampling and wildfires. However, these characters were systematically measured in all samples and used in the morphological description of the new species. After preliminary data manipulation, 31 of the original characters were used to perform the analyses.

A PCoA utilizing Gower’s distances (Gower 1971) was used to visualize the pattern of morphological variation among the studied taxa. Following the PCoA, a jackknifed canonical discriminant analysis (CDA) was performed (Krzanowski 1990), taking into account the morphological groups individuated with the PCoA and the ploidy levels inferred with flow cytometry. All characters invariant within the groups were excluded from the CDA dataset (bringing down the characters used to 25) as it is one of the fundamental assumptions to avoid any type of distortion in Discriminant Analyses.

All statistical analyses were computed with R (R Core Team 2024) in RStudio 2024.4.2.764 (Posit team 2024) using the MorphoTools2 package (Šlenker et al. 2022).

### ﻿Ploidy estimation

The ploidy level was measured in 50 samples (1–4 per population) using flow cytometry with DAPI dye. The youngest and most well-preserved leaves were selected from representative individuals of both fresh plants and herbarium vouchers (no older than one year). Samples were then co-chopped with the standard (*Lycopersicumesculentum* “Stupické polní tyčkové rané”) in a Petri dish containing 0.5 mL Otto I buffer (0.1M citric acid, 0.5% Tween 20; Otto 1990) using a razor blade. The nuclei suspension was then filtered through a 50 μm nylon mesh before 1 mL of Otto II buffer (0.4M Na2HPO4 · 12H2O) supplemented with 2 μg/mL DAPI was added. Samples were analyzed with a CyFlow ML flow cytometer (Partec GmbH, Germany) equipped with a UV light-emitting diode (365 nm, Sysmex Partec GmbH) at the Department of Botany and Zoology, Masaryk University, Brno, Czech Republic. To confirm the ploidy level, choromosome counts were performed using the Fuelgen protocol. In brief, Squash preparations were made on root tips obtained from germinating seeds. The root tips were pre-treated with 0.4% colchicine for 3 hours and then fixed in Carnoy fixative solution for 1 hour. After hydrolysis in HCl 1N at 60 °C for 7–8 minutes, the tips were stained in leuco-basic fuchsine for 3 hours.

### ﻿DNA extraction and ddRAD sequencing

Genomic DNA was extracted from silica gel-dried leaves or herbarium specimens from 14 samples of the same species included in the morphometric analyses (Suppl. material 1: table S7). DNA quality was assessed by 1.5% agarose gel electrophoresis, and concentration was measured using a Qubit 2 Fluorometer with the 1X dsDNA HS Assay Kit (Thermo Fisher Scientific).

The double digest restriction site-associated DNA (ddRAD) library preparation protocol was adapted from Sochor et al. (2024) with modifications. Briefly, 100 ng of genomic DNA was digested with SbfI-HF and MseI restriction enzymes in rCutSmart buffer (New England Biolabs) at 37 °C for 3 hours, followed by enzyme inactivation at 80 °C for 20 minutes. Immediately afterwards, P1 and P2 adapters, corresponding to the restriction sites of the respective enzymes, were ligated using T4 DNA ligase (New England Biolabs) at 16 °C overnight, with subsequent heat inactivation at 65 °C for 10 minutes. The P1 adapter for each sample contained a unique barcode for individual identification. The DNA concentration of each restricted sample was measured using the Qubit fluorometer, and equimolar amounts of digested DNA from all samples were pooled. The pooled samples were purified with 1.2 × SPRI magnetic beads and used as a template for PCR enrichment. PCR amplification was performed in four 20 µl reactions (18 cycles each) using Phusion HF PCR Mastermix (New England Biolabs) and standard Illumina P1-i5 (5’-AATGATACGGCGACCACCGA-3’) and P2-i7 (5’-CAAGCAGAAGACGGCATACGA-3’) primers. The protocol for each step is available in the Suppl. material 1: table S6. The final amplified products were size-selected using SPRI magnetic beads, with left and right side selections at 0.5 × and 0.9 × ratios, following the manufacturer’s protocol. The final ddRAD library was sequenced at the CEITEC facility (Brno, Czech Republic) on an Illumina NextSeq platform using a mid-output configuration with 300 cycles. Sequencing utilized a portion of the platform’s capacity, generating approximately 30,000,000 paired-end reads.

### ﻿Phylogenomic analysis

Paired-end reads generated by Illumina sequencing of the ddRAD library were demultiplexed and analyzed using iPyRAD v.0.9.97 (Eaton and Overcast 2020). Quality control parameters were configured as follows: trimming was performed for bases with a quality score below Q20, allowing up to five low-quality bases per read. The Phred Q score offset was set to 33. The minimum read depth for base calling and majority-rule consensus was set to 6, with a maximum read depth per sample capped at 10,000. The sequence similarity threshold was specified as 0.90, permitting a maximum of one base mismatch in barcodes. Adapter sequences were strictly filtered out, and a minimum read length of 35 bp was required. Consensus sequence assembly was conducted with the following parameters: a maximum of six alleles per consensus sequence to accommodate diploid and polyploid species in the dataset; a maximum of 5% uncalled bases and 5% heterozygous sites per consensus sequence; and a minimum of seven samples sharing data at a given locus (allowing up to 46% missing data). Locus filtering parameters were set to allow a maximum of 20% SNPs per locus, up to eight indels per locus, and a maximum of 20% heterozygous sites per locus.

Phylogenomic relationships among individuals based on ddRAD data were inferred using RAxML v.8.2.12 (Stamatakis 2014). The GTRCAT substitution model was employed, and bootstrap analysis with 1,000 replicates was performed to construct a maximum likelihood tree. The resulting phylogeny was visualized using FigTree v.1.4.4 (Rambaut 2015).

## ﻿Results

### ﻿Morphometric analysis

**Figure 1.**
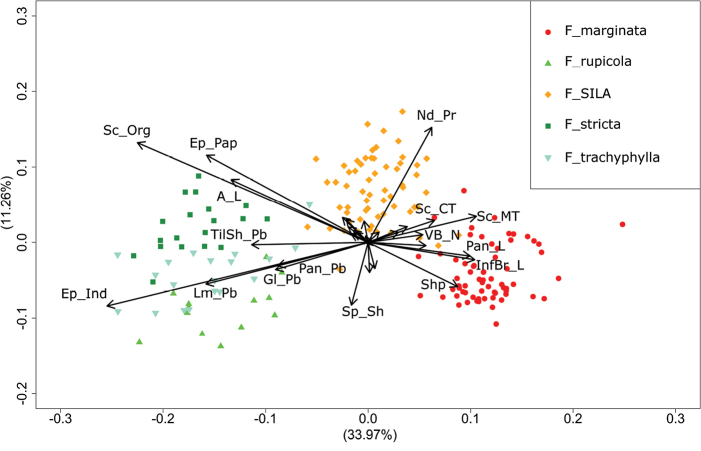
Biplot for the PCoA performed with Gower’s distance.

The first two axes of the exploratory PCoA account for 45.23% of the variability. Three main clusters can be observed in the biplot (Fig. 1): species of *F.stricta* group, the Sila hexaploid and the diploid *F.marginata*. The hexaploid taxon from the Sila plateau appears morphologically as an in-between entity but still clearly distinct from the other two groups. In particular, the taxon seems to share with the species of the *F.stricta* group the tendency to develop decurrent to confluent sclerenchyma strands (Sc_Org), as well as presenting undulations between epidermal cells (Ep_Pap) and longer awns (A_L). However, these are the only similarities between the two groups, as the Sila specimens resemble more, on a superficial level, species of the *F.marginata* group. They lack numerous other features typical of the *F.stricta* taxa, such as scabrid leaf blades due to the presence of barbs on the adaxial indumentum (Ep_Ind) and an overall pubescence observed in the panicle (Lm_Pb, Gl_PB, Pan_Pb) and tiller sheats (TilSh_Pb). Compared to the new species, *F.marginata* tends to develop discrete thickened sclerenchyma strands (Sc_Org, Sc_MT, Sc_CT), a larger number of vascular bundles (VB_N), bigger panicles(Pan_L, InfBr_L) and leaves in a more elongated conduplicate “V” shape (Shp). Finally, the Sila taxon seems to be overall a more pruinous plant (Nd_Pr, Sp_Sh) compared to all the other species analyzed. Based on the clustering of the taxa in the biplot, we decided to merge the species from the *Festucastricta* aggregate into a single group to avoid distortions in the following discriminant analysis.

**Figure 2.**
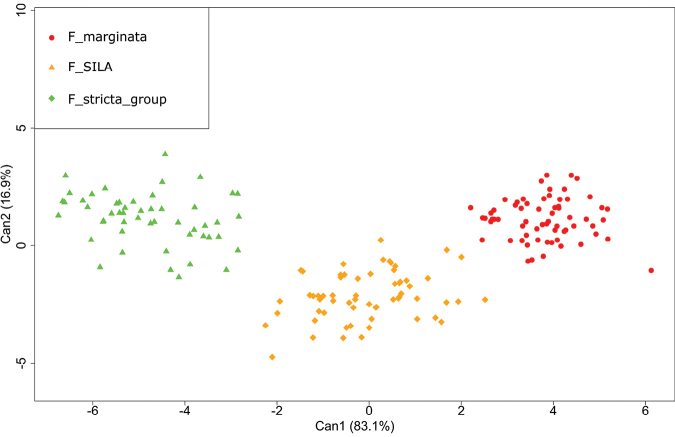
Scatterplot for the DA performed on the three morphological groups individuated by the PCoA.

The jackknifed CDA (Fig. 2) fully supports the grouping hypothesis indicated by the scatterplot of the PCoA with 97.74% classification success in 177 samples (Table 2). Again, the new species appears as a distinct morphological in-between entity of the other considered species.

**Table 2.** Confusion matrix for the DA performed on the three morphological groups individuated by the PCoA.

Taxon	N	*F.marginata*	*F.sila*	*F.stricta* group	correct	%
*F.marginata*	65	65	0	0	65	100.00
*F.silana*	61	3	58	0	58	95.08
*F.stricta* group	51	0	1	50	50	98.04
Total	177	68	59	50	173	97.74

### ﻿Ploidy estimation

Overall, 50 of the freshly collected individuals were studied with flow cytometry. All 20 individuals of *F.stricta* s.l. (*F.stricta*, *F.trachyphylla* and *F.rupicola*) were hexaploid and all *F.marginata* samples were diploid (17 individuals) in accordance with previous works (Portal 1999; Arndt 2008, Šmarda et al. 2008; Ardenghi et al. 2016, 2024). All 13 individuals assumed as *F.silana* were hexaploid.

### ﻿Phylogenomic analysis

**Figure 3.**
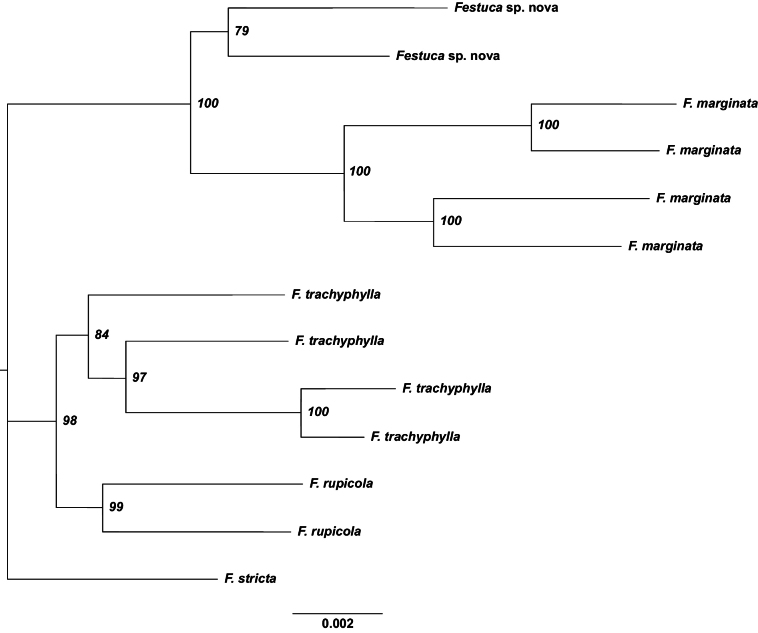
Phylogenetic tree of thirteen *Festuca* individuals, constructed using RAxML based on 2,713 loci and maximum likelihood estimation. The numbers in the nodes represent bootstrap values (%) from 1,000 replicates.

A total of 67,353 loci were initially identified, which were reduced to 2,713 after filtering. The primary cause of this reduction was the requirement for a minimum of seven out of 13 samples to contain data for a given locus. Following the first analysis, one sample (F14) with low sequencing coverage was excluded based on quality assessment, and the analyses were repeated without this sample. The number of retained loci per sample ranged from 1,526 to 2,096. The resulting best tree effectively resolved each species, with high bootstrap support for most nodes (Fig. 3).

### ﻿Taxonomic treatment

#### ﻿ Festuca silana

Taxon classificationPlantaePoalesPoaceae

Ardenghi, Pallanza & Foggi sp. nov

25CFCBE5-C028-504E-B9CA-FFE5D7FC927E

urn:lsid:ipni.org:names:77360044-1

##### Typus.

Italy • Sila piccola, Zagarise (Catanzaro), Latteria, Lato W del sentiero, Pendici SW del M. Gariglione (WGS84: 39°07'29.4"N, 16°37'32.8"E), 1587 m, prateria meso-xerofila con roccia granitica affiorante, assieme a *Patzkeapaniculata*, 06.07.2014, *N. Ardenghi & L. Bernardo*. (***holotype***: PAV-150000!; ***isotypes***: FI!, CLU!, W!, G!).

Italy • Sila grande, Spezzano della Sila (CS), Vaccarizzo, Sponda S del Lago Cecita (WGS84: 39°22'13.5"N, 16°30'40.1"E), 1150 m, prato arido con Astragalusparnassisubsp.clabricus, su sabbie granitiche con affioramenti rocciosi, 05.07.2023, *M. Pallanza & L. Bernardo*. (***paratypes***: PAV-150002!, PAV-150003!, FI!, CLU!, W!, G!).

Italy • Sila grande, Casali del Manco (CS), Lago Ariamacina, Sponda N del lago (WGS84: 39°20'00.8"N, 16°32'39.0"E), 1327 m, prato arido con Astragalusparnassisubsp.clabricus, su sabbie granitiche, 05.07.2023, *M. Pallanza & L. Bernardo*. (***paratypes***: FI!, CLU!, W!, G!).

Italy • Sila grande, San Giovanni in Fiore (CS), Carlomagno, A W della SP211 (WGS84: 39°16'58.1"N, 16°32'36.1"E), 1535 m, prato mesofilo a *Patzkeapaniculata*, su substrato granitico con spesso strato di suolo, 05.07.2023, *M. Pallanza & L. Bernardo*. (***paratypes***: PAV-150001!, FI!, CLU!, W!, G!).

##### Description.

**Figure 4.**
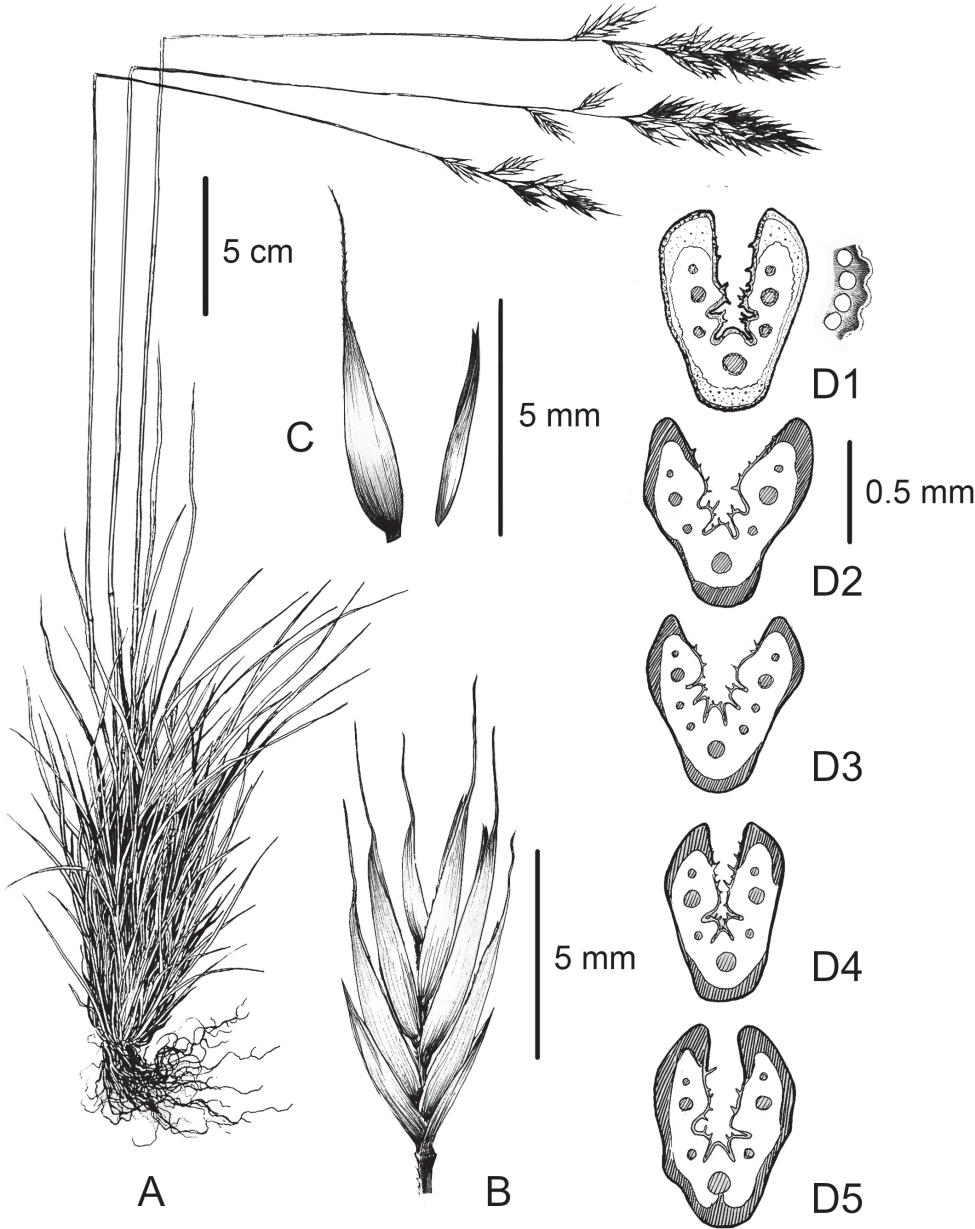
Illustration of *Festucasilana* Pallanza, Ardenghi & Foggi based on the specimens from the locus classicus **A** overall habitus and morphology of the species **B** detailed appearance of the spikelet **C** detailed view of lemma and palea **D1** leaf cross-section of the holotype **D2–D5** different leaves cross-sections showcasing intraspecific variability.

Perennial herb, caespitose to densely caespitose, occasionally pruinose. Tiller shoots intravaginal. Culms (32.0–) 44.7–57.2 (–69.5) cm long and (0.52–) 0.77–1.04 (–1.29) mm in diameter, erect cylindrical, canaliculated, smooth to the touch, with 1 (–2) visible nodes, dark brown to black in color, located in its lower third. Cauline leaves 1 (–2), (1.6–) 2.6–4.0 (–5.3) cm long, sheathing the culm for (5.8–) 8.0–10.3 (–12.8) cm. Basal leaf sheath glabrous, on extremely rare occasions with few sparse hairs, open down to the base, yellowish in color, more rarely veined in red. Ligula 0.5–0.8 mm long, membranaceous, truncate, fringed, with two auricles at the sides. Basal leaf blade (7–) 11.8–21.6 (–31.7) cm long and (0.75–) 0.94–1.06 (–1.36) mm in diameter, smooth, somewhat rigid, conduplicate, bright to dark green. Cross section outline in an open U-V shape. Subepidermal sclerenchyma organized in 3 main strands located at the margins and keel of the leaf blade, thickened and often decurrent or accompanied by secondary strands opposing vascular bundles, forming an interrupted or irregular complete ring. Vascular bundles 7(–9). Ribs 2 (–4). Abaxial surface of the leaf blade covered with a dense indumentum of (0.03–) 0.04–0.07 (–0.13) mm long hairs. Adaxial surface smooth, with small undulations between epidermal cells. Panicle (4–) 6.4–8.6 (–12.5) cm long, cylindrical to pyramidal during anthesis, dense, with 7–27 spikelets; branches 1–5, simple, antrorsely scabrid; nodes 7–12. Spikelets (6.24–) 7.33–8.13 (–8.87) long, laterally flattened, elliptic, green, with (3–) 4–6 (–8) fertile florets. Glumes 2, unequal, lanceolate, glabrous or rarely with few sparse hairs on the margins and apex. Lower glume (2.21–) 2.55–3.12 (–3.90) mm long, with a single nerve. Upper glume (2.46–) 3.68–4.31 (–5.12) mm long, 3-nerved. Lemma (3.82–) 4.81–5.26 (–5.83) mm long, lanceolate, glabrous or more rarely with few sparse hairs near the apex, terminating in an apical awn (1.23–) 1.83–2.52 (–3.30) mm long. Palea 4.53–5.71 mm long, lanceolate, bifid, with 2 finely dentated keels. Anthers 3, (1.72–) 2–2.44 (–2.93) mm long, yellow to orange in color. Ovary glabrous. Stygmas 2, with a feather-like shape. Lodicules 2, bilobed, 0.83–1.06 mm long. Caryopsis 2.42–3.85 mm long, brown to dark orange at maturity, adherent to the palea (Fig. 4). Somatic chromosomes 42 (2n = 6x).

##### Eponym.

The new species is named after the Sila plateau where it typically occurs.

##### Diagnosis.

Differt a *Festucacostei* (St.-Yves ex Litard.) Markgr.-Dann. crassiore sclerenchyma in laminarum margine et carina, plerumque 7 (non (7–) 9–11) fasciculis vascularibus, densioribus et longioribus trichomatibus in abaxiale superficie, minutis undulationibus inter epidermidis cellulas, 42 (non 28) chromosomatibus.

It differs from *Festucacostei* (St.-Yves ex Litard.) Markgr.-Dann. for the thicker sclerenchyma at margins and keel of the leaves, vascular bundles are rarely more than 7 (compared to the (7–) 9–11 in *F.costei*). Abaxial indumentum is more dense and with longer trichomes compared to *F.costei*. It also presents small undulations between epidermal cells. Somatic chromosomes 2n = 6x = 42 instead of 2n = 4x = 28 in *F.costei*.

Differt a *Festucamarginata* (Hack.) K.Richt. sclerenchyma plerumque decurrente vel completum anulum fingente potius quam in tribus discretis filis ordinato, fasciculis vascularibus raro plus quam 7 (non (7-) 9–11 ut solet in F.marginata), longioribus trichomatibus in densiore abaxiale superficie, longioribus aristis, undulationibus inter epidermidis cellulas, 42 chromosomatibus, non 14.

It differs from *Festucamarginata* (Hack.) K.Richt. in the sclerenchyma, usually decurrent or up to forming a complete ring instead of being organized in three discrete strands. Vascular bundles are rarely more than 7 (compared to the (7–) 9–11 in *F.marginata*). Abaxial indumentum is richer and with longer trichomes compared to *F.marginata*. Awns longer compared to *F.marginata*. Undulations are present in between epidermal cells. Somatic chromosomes 2n = 6x = 42 instead of 2n = 2x = 14 in *F.marginata*.

Differt a *Festucatrachyphylla* (Hack.) R.P.Murray sclerenchyma prope margines et carinam crassiore; foliis numquam scabris et colore viridi clariore (non glauco ut solet in *F.trachyphylla*); spiculis numquam pubescentibus; foliarum vaginis quam saepissime glabris (non pubescentibus ut solet in *F.trachyphylla*).

It differs from *Festucatrachyphylla* (Hack.) R.P.Murray in the sclerenchyma, thicker in the correspondence of margins and keel, the leaf blades never scabrid and of a brighter green color (opposed to the glaucus of *F.trachyphylla*). Spikelets are never pubescent. Leaf sheaths are glabrous except for extremely rare exceptions (opposed to the commonly pubescent in *F.trachyphylla*).

##### Distribution and ecology.

**Figure 5.**
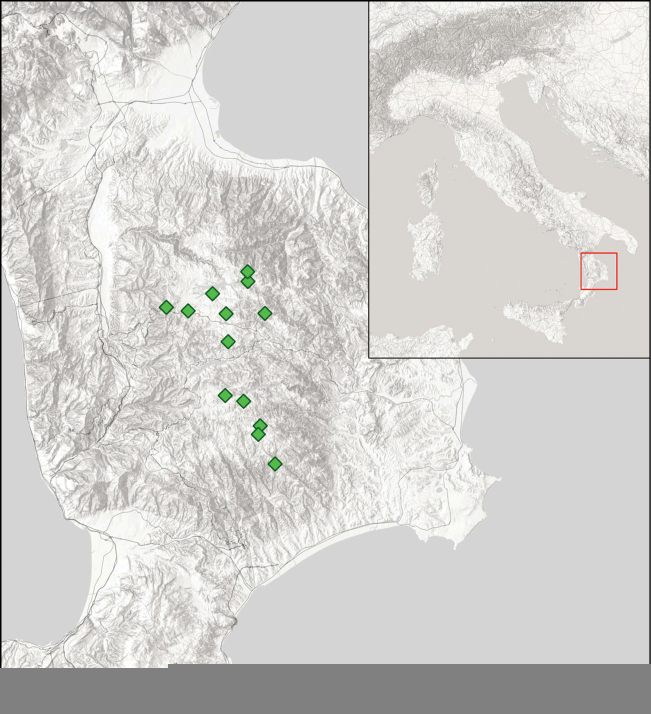
Map of the Sila and known stations of *F.silana* occurring within the plateau.

The species is only known from the Sila highland, currently from 13 populations (Fig. 5). It mainly grows in grassland and pasture communities (*Anthemidetaliacalabricae* Brullo, Scelsi & Spampinato, 2001) on granitic sands in xerophilic to mesophilic conditions at altitudes between 1100 m and 1600 m. *F.silana* typically occurs in grasslands of xerophile to mesophile conditions, along with other *Festuca* taxa such as F.marginatasubsp.marginata, *F.trachyphylla*, F.rubrasubsp.commutata and *F.cyrnea*.

##### Conservation status.

Although the plant has a distribution limited to the Sila plateau, it is one of the dominant species in the grasslands that it inhabits and lacks any particular threat that could cause a decline in the population. Therefore, it had to be considered as Least Concern (LC) according to IUCN (2012).

### ﻿Key to the studied species

**Table:** 

1	Sclerenchyma strands decurrent sometimes with accessory strands or confluent in an irregular complete ring. Spikelets 7–8.5 mm. Awns usually > 2 mm long. Hexaploid plants	**2**
–	Sclerenchyma in three discrete strands, never decurrent. Spikelets 6–7 mm. Awns short, generally < 2 mm. Diploid plants.	**F.marginatasubsp.marginata**
2	Leaf scabrid, at least in the apicat/upper part. Spikelets and leaf sheaths generally pubescent. Plants glaucous to dark green.	**3**
–	Leaves completely glatt/smooth, never scabrid. Spikelets glabrous. Leaf sheaths usually glabrous, only very rarely with sparse hair. Plant bright/fresh green.	***F.silana***
3	Tiller leaves with 5(rarely 7) vascular bundles. Sclerenchyma of regular thickness throughout its length. Plants of natural areas.	**4**
–	Tiller leaves with 7–9 (rarely 5) vacular bundles. Sclerenchyma irregularly thickened throughout its length. Plant typical of synantropic or disturbed habitats.	***F.trachyphylla***
4	Sclerenchyma generally forming a thick continuous ring, more rarely partly interrupted. Leaves strongly scabrid.	***F.stricta***
–	Sclerenchyma in three decurrent strands at the margins and keel, more rarely with accessory strands opposed to the vascular bundles. Leaves moderately to weakly scabrid.	***F.rupicola***

## ﻿Discussion

Despite the superficial similarities with other taxa of the *Festucamarginata* group and *F.trachyphylla*, our findings fully support *F.silana* as a standalone species. Attempts to identify the samples of *F.silana* with current keys of the Italian flora led to *F.costei* due to the high importance of the sclerenchyma ring and the general appearance of plants resembling the *F.marginata* group. However, many morphological characters neglected by the keys (such as the number of vascular bundles, the presence of undulations in-between epidermal cells, shorter arms of the leaf cross-section, etc.) easily emerge when looking at the specimens of the two taxa side by side. *F.costei* (= *F.arvernensis* Auquier, Kerguélen & Markgr.-Dann. subsp. costei (St.-Yves) Auquier & Kerguélen) has been described for the Massif Central in France and never reported for the Apennines. The separation between the two taxa was also confirmed by the ploidy inference via flow cytometry. *F.silana* is always hexaploid, thereby excluding any possibility of it being a particular morphotype of *F.costei*, which has been consistently reported as tetraploid (Auquier and Kerguélen 1977; Portal 1999; Šmarda et al. 2008). Additionally, although *F.costei* appears in the most recent checklists of the Italian vascular flora (Bartolucci et al. 2018, 2024), it has been reported for a single locality in the Maritime Alps (Piedmont, Italy; Foggi et al. 2017), but we could not trace any herbarium specimens supporting this data. Moreover, we visited the locality reported by Foggi et al. (2017) and could not find any individuals of this taxon here. Finally, biogeographical considerations were quite weak in supporting this determination and a disjunction between Massif Central – Southern Apennines is not very plausible. In light of these consideration, *F.costei* is not a component of the flora of Italy.

Despite the shared hexaploidy and similar leaf cross-sections’ anatomy, fescues of the *F.stricta* group differ from *F.silana* in their commonly pubescent tiller leaves’ sheaths and spikelets. Also, species of the *F.stricta* group commonly display some level of scabridity in the tiller leaf blades, which never occurs in *F.silana*. Finally, *F.silana* differs from *F.marginata* from the strong morphological distinction in leaf cross section anatomy, especially in sclerenchyma development and structure, as well as for the ploidy level (diploid vs hexaploid). The morphological differences can be attributed to the different ploidy levels, as it has been shown that an increase in ploidy level influences different morphological characters in fine-leaved fescues (Rewicz et al. 2018).

It is reasonable to think that *F.silana* may have originated from autopoliploidy of *F.marginata*, which is also present in the Sila plateau and sometimes even within the same localities. This hypothesis is supported by the ddRADseq sequencing results. It should be noted that the *F.marginata* group has recently been revised in Central-Southern Europe (Ardenghi et al. 2024) and all the previously described taxa have been reduced to two single diploid subspecies reflecting major differences in morphology and genome size between the populations in Greece (considered as Festucamarginatasubsp.heldreichii (Hack.) Ardenghi & Foggi) and the remaining European populations (reported under Festucamarginata(Hack.)K. Richt.subsp.marginata). This newly discovered taxa from the Southern Apennines highlights the need for continued taxonomic research to broaden the understanding of plant diversity of critical groups such as *Festuca* in the Apennines.

## Supplementary Material

XML Treatment for Festuca silana

## ﻿Additional information

### Author ORCIDs

Mattia Pallanza https://orcid.org/0009-0006-7734-6191

Simone Orsenigo https://orcid.org/0000-0003-0348-9115

Nicola Maria Giuseppe Ardenghi https://orcid.org/0000-0002-4366-5729

Liliana Bernardo https://orcid.org/0000-0002-6703-6409

Petr Šmarda https://orcid.org/0000-0003-1048-9199

Petra Šarhanová https://orcid.org/0000-0002-1006-4003

Graziano Rossi https://orcid.org/0000-0002-5102-5019

Bruno Foggi https://orcid.org/0000-0001-6451-4025

### Data availability

All of the data that support the findings of this study are available in the main text or Supplementary Information.

## Supplementary materials

Supplementary material 1 Occurrences, studied specimens, protocols Data type xlsx Explanation note Excel file with a guide sheet describing the content of the different other sheets. A List of herbaria specimens studied as an exploratory part of the research, datasets for the PCoA and DA, Protocols and information on the samples used in the DNA extraction and ddRAD sequencing are available. https://binary.pensoft.net/file/1304720This dataset is made available under the Open Database License (http://opendatacommons.org/licenses/odbl/1.0/). The Open Database License (ODbL) is a license agreement intended to allow users to freely share, modify, and use this Dataset while maintaining this same freedom for others, provided that the original source and author(s) are credited.Mattia Pallanza, Orsenigo Simone, Petr Šmarda, Petra Šarhanová
